# ROCK1 via LIM kinase regulates growth, maturation and actin based functions in mast cells

**DOI:** 10.18632/oncotarget.7851

**Published:** 2016-03-02

**Authors:** Reuben Kapur, Jianjian Shi, Joydeep Ghosh, Veerendra Munugalavadla, Emily Sims, Holly Martin, Lei Wei, Raghuveer Singh Mali

**Affiliations:** ^1^ Department of Pediatrics, Herman B Wells Center for Pediatric Research, Indiana University School of Medicine, Indianapolis, IN, USA; ^2^ Department of Microbiology and Immunology, Indiana University School of Medicine, Indianapolis, IN, USA; ^3^ Gilead Sciences, Inc., Foster City, CA, USA

**Keywords:** mast cells, Rho kinase, LIM kinase, cell growth, anaphylaxis

## Abstract

Understanding mast cell development is essential due to their critical role in regulating immunity and autoimmune diseases. Here, we show how Rho kinases (ROCK) regulate mast cell development and can function as therapeutic targets for treating allergic diseases. Rock1 deficiency results in delayed maturation of bone marrow derived mast cells (BMMCs) in response to IL-3 stimulation and reduced growth in response to stem cell factor (SCF) stimulation. Further, integrin-mediated adhesion and migration, and IgE-mediated degranulation are all impaired in Rock1-deficient BMMCs. To understand the mechanism behind altered mast cell development in Rock1−/− BMMCs, we analyzed the activation of ROCK and its downstream targets including LIM kinase (LIMK). We observed reduced activation of ROCK, LIMK, AKT and ERK1/2 in Rock1-deficient BMMCs in response to SCF stimulation. Further, loss of either Limk1 or Limk2 also demonstrated altered BMMC maturation and growth; combined deletion of both Limk1 and Limk2 resulted in further reduction in BMMC maturation and growth. In passive cutaneous anaphylaxis model, deficiency of Rock1 or treatment with ROCK inhibitor Fasudil protected mice against IgE-mediated challenge. Our results identify ROCK/LIMK pathway as a novel therapeutic target for treating allergic diseases involving mast cells.

## INTRODUCTION

Mast cells regulate both innate and adaptive immunity and are involved in various autoimmune diseases [1–3]. KIT and IL-3 receptor signaling plays a critical role in mast cell growth and differentiation [4–8]. In addition to KIT and IL-3 receptors, mast cells also constitutively express the high-affinity receptor for Immunoglobulin E (IgE), FcεRI [1, 9]. IgE-mediated mast cell activation causes mast cell degranulation leading to release of inflammatory mediators causing allergic reaction [1, 9]. Evidence suggests that IgE-dependent activation of mast cells is critical for the pathophysiological manifestations and mortality associated with IgE-dependent anaphylaxis [1, 9]. Further, mast cells are also major effector cells of asthma and immune responses to parasites [10, 11]. Recent studies show that many asthma patients have sensitivity to environmental allergens and increased levels of antigen-specific and total IgE antibodies in serum [12, 13]. Although involvement of mast cells in allergic diseases is well characterized, this knowledge has not been translated into new therapeutics in part to inadequate understanding of the signaling pathways involved in the development of mast cells.

Rho kinases or Rho-associated coiled coil-containing protein kinases (ROCK) are protein serine/threonine kinases. Two isoforms of ROCK have been described which are encoded by two separate genes, Rock1 and Rock2 [14]. Rock1 and Rock2 share considerable sequence homology at the protein level; close to 65% overall and nearly 92% in their kinase domains [14]. Activation of ROCK leads to phosphorylation of multiple target proteins including LIM kinases (LIMK) [15, 16], myosin phosphatase (MYPT1) [17], and myosin light chain (MLC) [18], which result in the recruitment of mediators of actin polymerization and formation of focal adhesions leading to changes in growth, survival and cell motility [19]. LIM kinases (LIMK) are serine/threonine kinases containing two N-terminal LIM domains, a PDZ domain and a C-terminal kinase domain [20–22]. There are two isoforms of LIMK, Limk1 and Limk2 which share 50% identity overall in amino acid sequence and 70% homology in kinase domain [20–22]. LIMK regulates actin dynamics through phosphorylation and inactivation of cofilin, a member of the actin depolymerizing factor (ADF) family [23, 24]. Although the role of ROCK/LIMK pathway in regulating actin dynamics is well known, their role in mast cell development and functions is not known. Here, we define how ROCK/LIMK pathway regulates mast cell development and functions, and identify ROCK inhibitors as therapeutic drugs for allergic diseases.

## RESULTS

### Deficiency of Rock1 results in impaired early maturation of bone marrow derived mast cells (BMMC)

**Figure 1:**
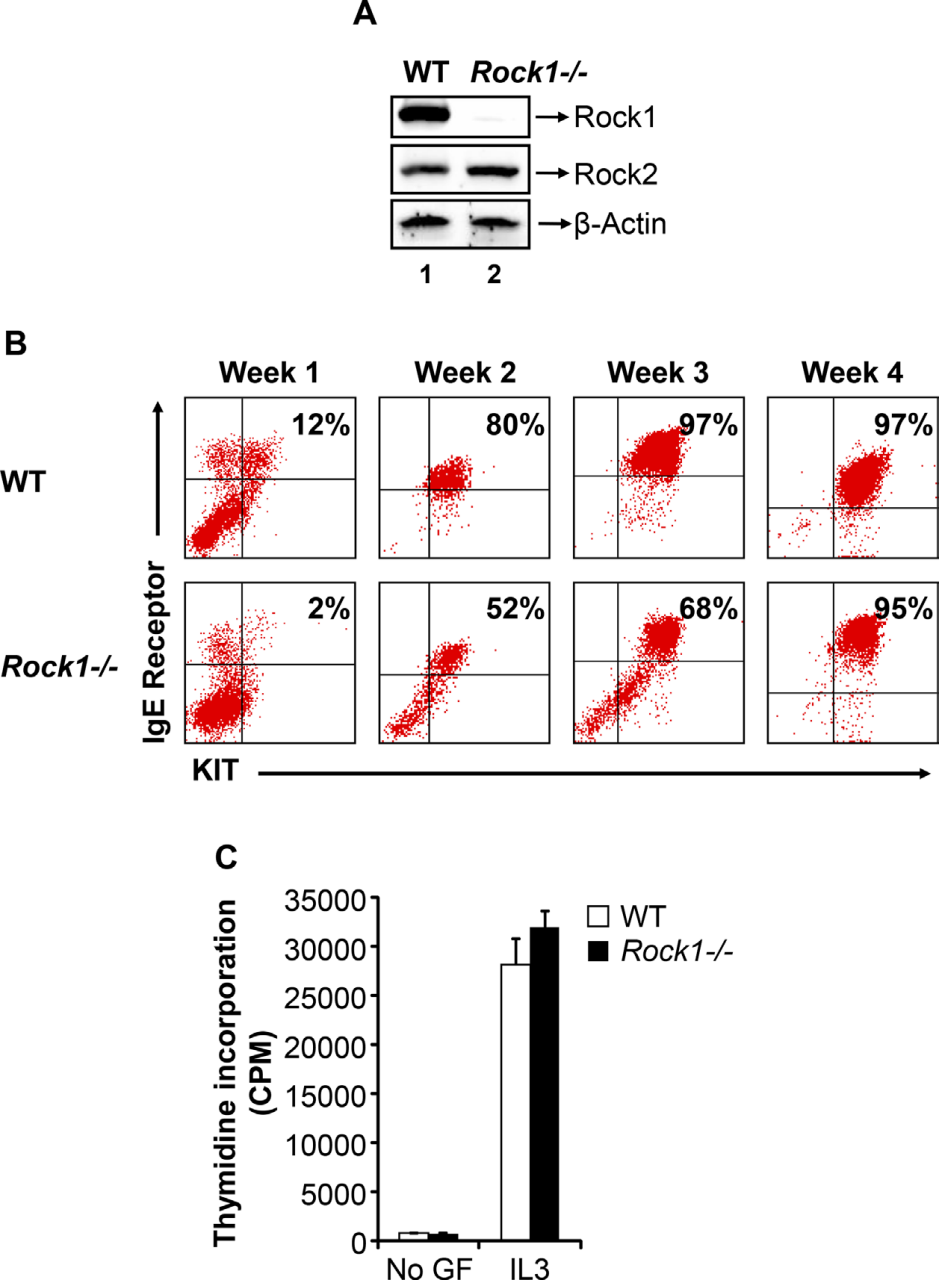
Deficiency of Rock1 results in impaired early maturation of bone marrow derived mast cell (BMMC) (**A**) Expression of ROCK isoforms in WT and *Rock1*−*/*− BMMCs. Equal amount of protein lysates from WT and *Rock1*−*/*− BMMCs were subjected to western blot analysis using a Rock1-specific antibody, Rock2-specific antibody and β-actin antibody as indicated. Expression of ROCK isoforms is indicated. (**B**) Deficiency of Rock1 delays the maturation of BMMCs. LDMNCs from WT and *Rock1−/−* mice were cultured in the presence of IL-3 (10 ng/mL) for 4 weeks. At indicated time points, maturation was analyzed by staining the cells with antibodies that recognize KIT and IgE receptor followed by flow cytometry. Shown is dot blot profile from one of three independent experiments. (**C**) Rock1 deficiency have no effect on IL-3 mediated growth of BMMCs. BMMCs from WT and *Rock1−/−* mice were starved for 6 hours in serum- and cytokine-free media and cultured in the presence or absence of IL-3 (10 ng/mL). After 48 hours, proliferation was evaluated by [^3^H] thymidine incorporation. Bars represent the mean [^3^H] thymidine incorporation in BMMCs (CPM + SD) from one representative experiment performed in quadruplicate. Similar results were observed in three independent experiments.

To determine the role of ROCK in mast cell maturation from its precursors in the bone marrow (BM), we utilized BM cells derived from WT and *Rock1−/−* mice. Figure 1A shows deletion of Rock1 and expression of Rock2 in *Rock1−/−* bone marrow derived mast cells (BMMC). BM cells from WT and *Rock1−/−* mice were cultured for 4 weeks in the presence of IL-3 and their maturation into mast cells was assessed by staining the cells for the co-expression of KIT and FcεR1 receptors followed by flow cytometric analysis. While WT BM cells fully matured into KIT and IgE receptor double positive mast cells by three weeks of culture, Rock1-deficient BMMCs showed reduced maturation at early time points, however their maturation was complete by 4 weeks as assessed by the presence of close to 100% KIT and IgE receptor double positive cells (Figure 1B). Thus, although *Rock1−/−* BM cells lag behind their WT counter parts with respect to the acquisition of KIT and IgE receptor double positive BMMCs, their expression is completely attained by the end of the 4th week of culture. We next analyzed the growth of WT and *Rock1−/−* BMMC's in response to IL-3 by thymidine incorporation. As seen in Figure 1C, no difference in the growth of WT and *Rock1−/−* BMMCs was observed in the presence of IL-3. These results suggest that Rock1 may play a minor role in the differentiation of BMMCs from its precursors in the BM.

### Rock1 deficient BMMCs show reduced growth in response to SCF

**Figure 2:**
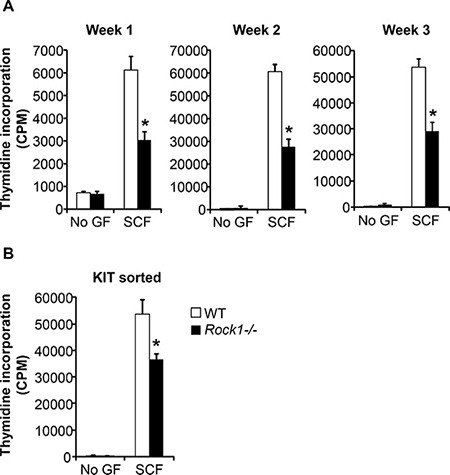
Rock1 deficient cells show altered SCF-mediated growth (**A**) Deficiency of Rock1 alters the SCF-mediated growth of BMMCs. LDMNCs from WT and *Rock1−/−* mice were cultured in the presence of IL-3 (10 ng/mL) for 3 weeks. At indicated time points, proliferation was evaluated by [^3^H] thymidine incorporation. BMMCs from WT and *Rock1−/−* mice were starved for 6 hours in serum- and cytokine-free media and cultured in the presence or absence of SCF (50 ng/mL). After 48 hours, proliferation was evaluated by [^3^H] thymidine incorporation. Bars represent the mean [^3^H] thymidine incorporation in BMMCs (CPM + SD) from one representative experiment performed in quadruplicate. Similar results were observed in three independent experiments. *p < 0.05, WT *vs*. *Rock1−/−*. (**B**) Reduced growth of KIT positive Rock1 deficient BMMMs. LDMNCs from WT and *Rock1−/−* mice were cultured in the presence of IL-3 (10 ng/mL) for 3 weeks. After 3 weeks, BMMCs were sorted based on KIT expression. KIT positive cells were starved for 6 hours in serum- and cytokine-free media and cultured in the presence or absence of SCF (50 ng/mL). After 48 hours, proliferation was evaluated by a [^3^H] thymidine incorporation assay. Bars represent the mean [^3^H] thymidine incorporation in BMMCs (CPM + SD) from one experiment performed in quadruplicate. *p < 0.05, WT *vs*. *Rock1−/−*.

We next performed studies to analyze the role of Rock1 in KIT receptor mediated growth of BMMCs. BMMCs derived from WT and *Rock1−/−* mice at the end of week 1, week 2 and week 3 were starved and cultured in the presence or absence of SCF for 48 hours, and proliferation was analyzed by thymidine incorporation. While WT BMMCs demonstrated a significant increase in thymidine incorporation in the presence of SCF relative to un-stimulated cells, deficiency of Rock1 resulted in a significant loss of SCF-mediated growth (Figure 2A). The reduced SCF-mediated growth was noted at the end of week 1, week 2 and week 3. Since Rock1 deficient cells show reduced maturation at early times during the culture period, we further assessed if the reduced growth in response to SCF is either due to reduced KIT expression or due to a cell intrinsic defect as a result of Rock1 deficiency. We sorted WT and *Rock1−/−* BMMCs based on KIT expression and measured growth in response to SCF stimulation by thymidine incorporation. As seen in Figure 2B, sorted KIT positive *Rock1−/−* BMMCs also showed reduced growth in response to SCF suggesting a critical role for Rock1 in normal growth of mast cells.

### Impaired adhesion and migration of BMMCs lacking the expression of Rock1

**Figure 3:**
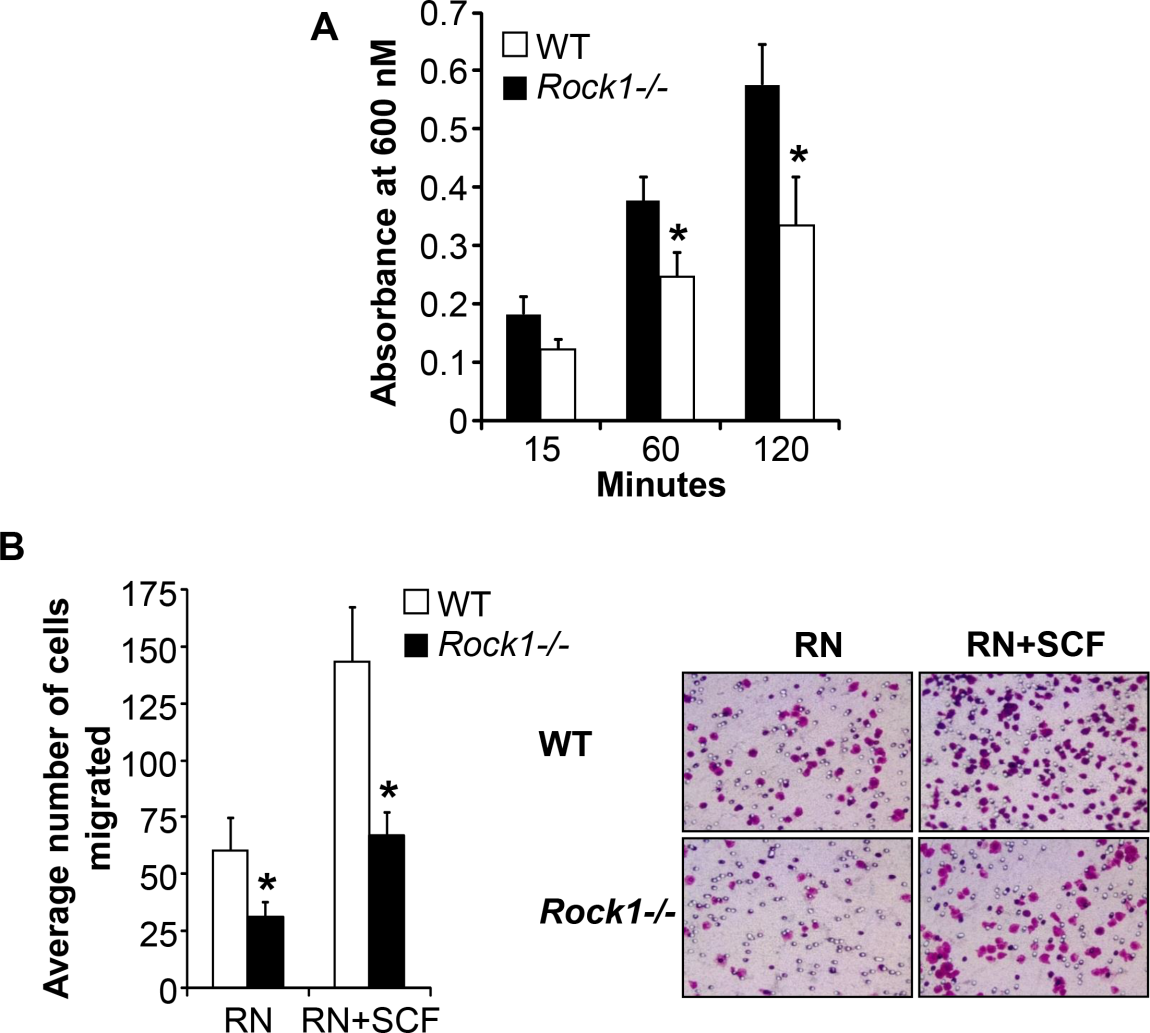
Rock1 deficiency impacts integrin-mediated adhesion and migration (**A**) BMMC's from WT or *Rock1−/−* mice (1 × 10^5^) were subjected to an *in vitro* adhesion assay on retronectin as described in methods. Adhesion was assessed by measuring absorbance at 600 nM at indicated times. Shown is pooled data from 4–6 independent experiments performed in triplicates ± SEM. *P < 0.05 for WT *vs Rock1−/−*. (**B**) BMMC's from WT or *Rock1−/−* mice (2.5 × 10^5^) were subjected to an *in vitro* migration assay on retronectin as described in methods. Cell migration was performed either in the presence or absence of SCF (50 ng/mL) and is expressed as the average number of cells migrated ± SD. Ten fields were scored from one representative experiment. Similar results were observed in 4 independent experiments. *P < 0.05, WT *vs Rock1−/−*.

Cellular adhesion is mediated by the direct ligation of integrin extracellular domains to defined sequences within the extracellular matrix. To understand the physiologic role of Rock1 in integrin-mediated adhesion, we performed adhesion assays on fibronectin using BMMCs derived from WT and *Rock1*−/− mice. As seen in Figure 3A, deficiency of Rock1 in BMMCs resulted in a significant reduction in adhesion of BMMCs to fibronectin at different time points tested. Since ROCK also regulate integrin-mediated migration, we next examined the role of Rock1 in integrin (haptotactic) as well as in cytokine-induced (chemotaxis) migration of BMMCs. BMMCs deficient in Rock1 showed a significant reduction in directional migration on retronectin alone and in combination with SCF and retronectin (Figure 3B). These results suggest that Rock1 plays a vital role in integrin-mediated adhesion and migration of BMMCs.

### Deficiency of Rock1 results in reduced IgE-induced degranulation of BMMCs

**Figure 4:**
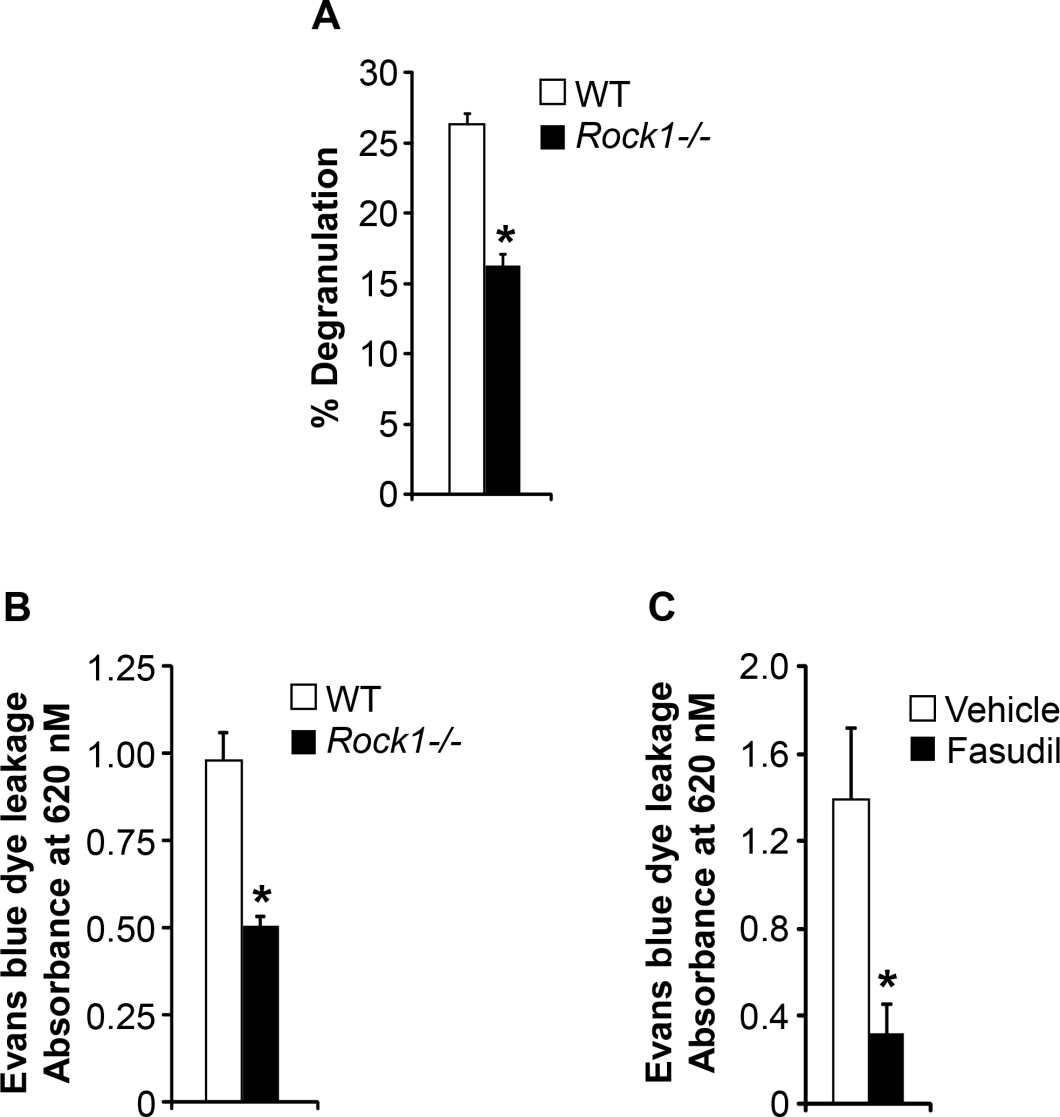
ROCK inhibition protects against passive cutaneous anaphylaxis (PCA) (**A**) Deficiency of Rock1 causes reduced mast cell degranulation. BMMC's from WT or *Rock1−/−* mice were subjected to an *in vitro* degranulation assay as described in methods. The degree of degranulation was determined by measuring the release of β-hexosaminidase. Shown is percentage degranulation ± SD from one representative experiment performed in quadruplicates. Similar results were observed in 4 independent experiments. *P < 0.05, WT *vs Rock1−/−*. (**B**) WT and *Rock1−/−* mice were injected intradermally with 20 ng anti-DNP IgE on the left ear, whereas the right ear received saline as a control. After 24 hours, mice received 100 μg DNP-BSA containing Evan's blue dye (1% wt/vol) via tail vein injection. After 30 minutes, ear punches (8 mm) of both ears were collected and were used for extraction of the Evan's blue dye followed by measurement of absorbance at 620 nm. Data is mean ± SEM from one independent experiment (*n* = 3 mice in each group). *P < 0.05 WT *vs Rock1−/−* mice. Similar results were observed in two independent experiments. Right panel shows the photographs of ears received anti-DNP IgE. (**C**) WT mice were injected intradermally with 20 ng anti-DNP IgE on the left ear, whereas the right ear received saline as a control. After 24 hours, mice were divided into two groups and one group received PBS where as other group received ROCK inhibitor fasudil (25 mg/kg body weight). After 30 minutes of drug treatment, mice received 100 μg DNP-BSA containing Evan's blue dye (1% wt/vol) via tail vein injection. After 30 minutes, ear punches (8 mm) of both ears were collected and were used for extraction of the Evan's blue dye followed by measurement of absorbance at 620 nm. Data is Mean ± SEM from one independent experiment (*n* = 3 mice in each group). *P < 0.05 vehicle *vs* fasudil treated.

Mast cells become activated when surface receptor-bound antigen-specific immunoglobulin E (IgE) encounters an antigen that the IgE recognizes. This triggers mast-cell degranulation leading to the rapid release of inflammatory mediators including histamine, proteoglycans, and cytokines [9, 25, 26]. To understand the role of Rock1 in mast cell functions, we performed degranulation assay in response to IgE stimulation using BMMCs derived from WT and *Rock1−/−* mice. As seen in Figure 4A, deficiency of Rock1 resulted in a significant reduction in IgE-induced degranulation. Ag- and IgE-dependent activation of mast cells via aggregation of FcεRI is critical for IgE-dependent anaphylaxis. Since we observed reduced mast cell activation and degranulation in *Rock1−/−* BMMCs, we next wanted to determine whether Rock1 plays any role in anaphylaxis. For this, we used an anaphylaxis mouse model *in vivo* in which IgE-induced passive cutaneous anaphylaxis (PCA) is elicited by injecting mice systemically with IgE antibodies 24 hours before an intravenous challenge with a specific antigen [27]. As seen in Figure 4B, mice injected with IgE showed significantly profound allergic reaction compared to PBS injected mice (data not shown). Importantly, loss of Rock1 resulted in significantly reduced allergic reaction compared to control mice (Figure 4B). Consistent with these findings, treating the mice with a ROCK inhibitor, fasudil, significantly inhibited this process (Figure 4C). Taken together these results suggest that Rock1 plays an important role in mast cell activation and ROCK inhibitors might act as therapeutic targets for treating allergic diseases involving mast cells.

### Reduced activation of ROCK and its downstream targets LIMK, AKT and ERK MAP kinase in *Rock1−/−* BMMCs in response to SCF

**Figure 5:**
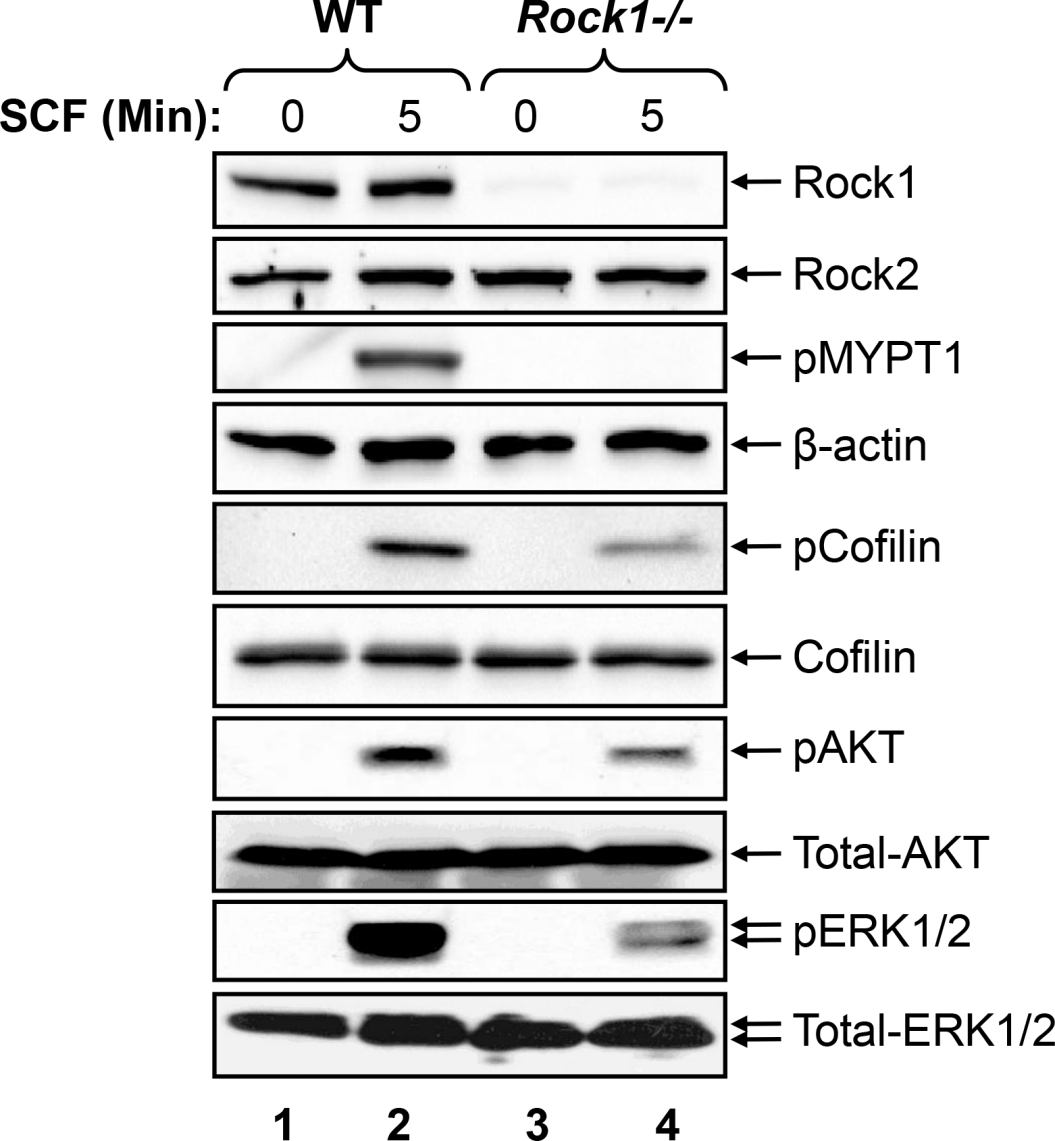
Rock1 regulates KIT mediated signaling in BMMCs Reduced activation of ROCK, LIMK, AKT and ERK MAP kinase in Rock1-deficient BMMCs. WT and *Rock1−/−* BMMCs were starved overnight in serum- and cytokine-free media and stimulated with SCF (100 ng/mL) for 5 min. Equal amount of protein lysates were subjected to western blot analysis using an anti-Rock1, anti-Rock2, anti-phospho-MYPT1, anti-β-actin, anti-phospho-AKT, anti-phospho-ERK1/2, anti-total AKT or anti-total ERK1/2 antibodies as indicated. Similar findings were observed in 3–4 independent experiments.

To determine the mechanism behind altered mast cell development and functions in *Rock1−/−* BMMCs, we analyzed the activation of ROCK and its downstream targets LIMK, PI3Kinase/AKT and ERK MAP kinase in WT and *Rock1−/−* BMMCs. BMMCs from WT and *Rock1−/−* mice were starved and stimulated with SCF for 5 min and analyzed for the activation of ROCK, LIMK, AKT and ERK by Western blotting. ROCK activation was determined by analyzing the phosphorylation of myosin phosphatase, MYPT1, which is downstream target of ROCK. Similarly, LIMK activation was determined by analyzing the phosphorylation of its downstream target cofilin. As seen in Figure 5, a significant decrease in the activation of ROCK, LIMK, AKT and ERK1/2 was observed in *Rock1−/−* BMMCs relative to WT. Thus, differential regulation of BMMC growth is likely due to differential activation of LIMK, AKT and ERK MAP kinase downstream from KIT.

### ROCK downstream effector LIMK is involved in mast cell differentiation and growth

**Figure 6:**
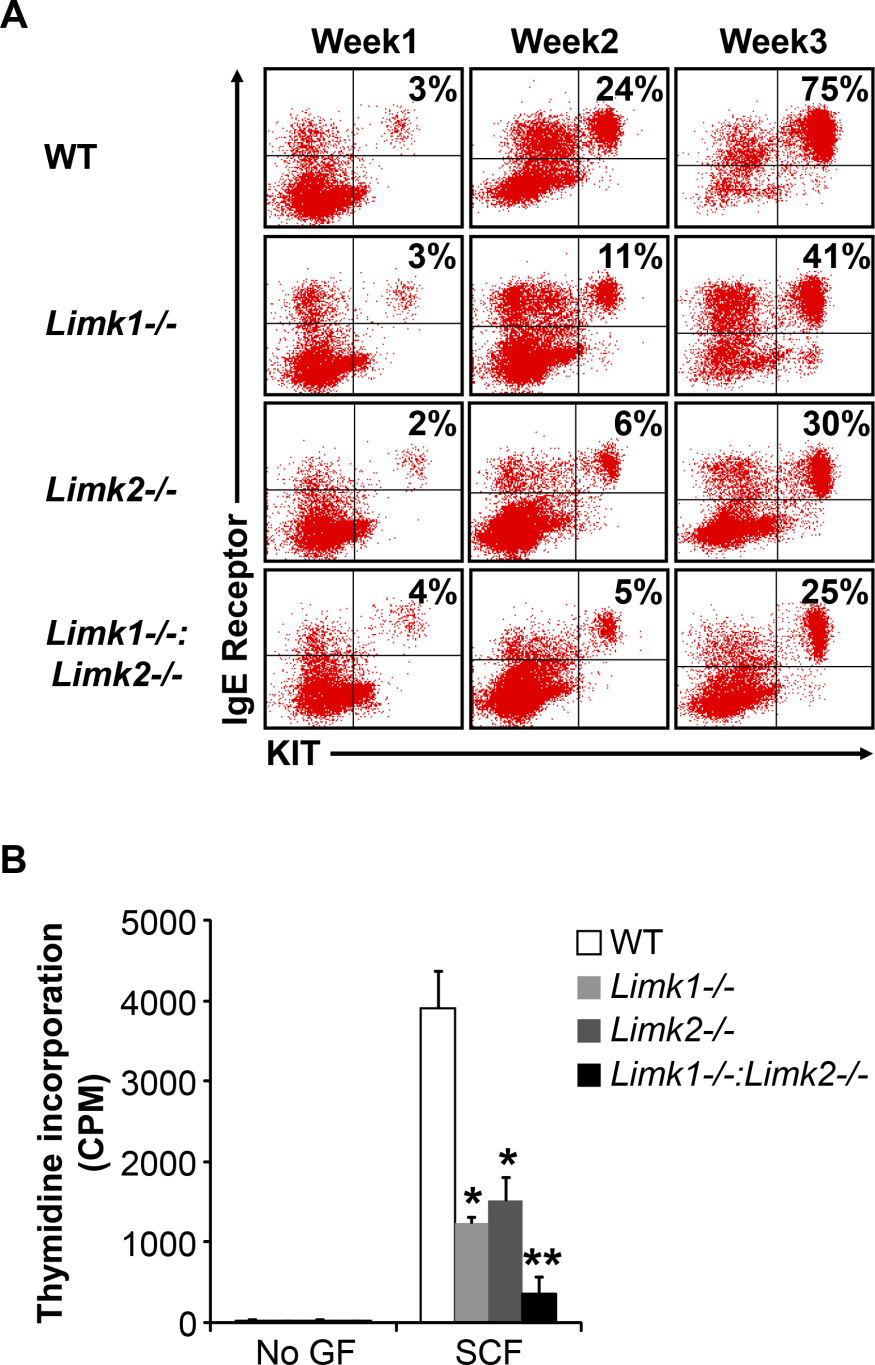
LIMK plays an important role in BMMC maturation, growth and survival (**A**) LDMNCs from WT, *Limk1*−*/*−*, Limk2*−*/*− and *Limk1*−*/*−*:Limk2*−*/*− mice were cultured in the presence of IL-3 (10 ng/mL) for 3 weeks. At indicated time points, maturation was analyzed by staining the cells with antibodies that recognize KIT and IgE receptor followed by flow cytometry. Shown is dot blot profile from one of four independent experiments. (**B**) BMMCs from WT, *Limk1*−*/*−*, Limk2*−*/*− and *Limk1*−*/*−*:Limk2*−*/*− mice were starved for 6 hours in serum- and cytokine-free media and cultured in the presence or absence of SCF (50 ng/mL). After 48 hours, proliferation was evaluated by [^3^H] thymidine incorporation. Bars represent the mean [^3^H] thymidine incorporation in BMMCs (CPM + SD) from one representative experiment performed in triplicate. Similar results were observed in four independent experiments. *p < 0.05, WT *vs. Limk1*−*/*− or *Limk2*−*/*−, **p < 0.05, *Limk1*−*/*− or *Limk2*−*/*− *vs. Limk1*−*/*−*:Limk2*−*/*−.

We next studied the role of LIMK in mast cell differentiation and growth to determine whether the altered mast cell development and functions in *Rock1−/−* BMMCs is due to reduced activation of LIMK. To study the role of LIMK in mast cell development, we used *Limk1−/−*, *Limk2−/−* and *Limk1/−/−:Limk2−/−* mice. We first cultured BM cells from WT, *Limk1−/−*, *Limk2−/−* and *Limk1/−/−:Limk2−/−* mice in the presence of IL-3 (10 ng/mL) for 3 weeks and at the end of week 1, week 2 and week 3 measured mast cell differentiation by analyzing the co-expression of KIT and FcεR1 receptors by flow cytometry. As seen in Figure 6, BMMCs from *Limk1−/−*, *Limk2−/−* and *Limk1/−/−:Limk2−/−* mice show reduced maturation at the end of week 2 and week 3 compared to WT BMMCs. Mast cell differentiation is further less in *Limk1/−/−:Limk2−/−* BMMCs compared to *Limk1−/−* or *Limk2−/−* BMMCs. We next analyzed the growth of WT, *Limk1−/−*, *Limk2−/−* and *Limk1/−/−:Limk2−/−* BMMCs in the presence of SCF. While loss of Limk1 or Limk2 alone shows significantly reduced growth of BMMCs in response to SCF stimulation compared to WT BMMCs, combined loss of both Limk1 and Limk2 shows further reduction in SCF-mediated growth (Figure 6B). These results suggest that LIMK plays a major role in mast cell differentiation and growth.

## DISCUSSION

Mechanisms involved in the growth, maturation and function of bone marrow derived mast cells have not been fully characterized. In the current study, we have explored the role of ROCK1 and Limk1/2 in the maturation, growth and actin based functions in bone marrow derived mast cells. Our results show that both Rock1 and Rock2 are expressed in bone marrow derived mast cells, the expression of Rock2 is not modulated in the absence of Rock1 in these cells and the phenotypic changes observed in Rock1 deficient mast cells are seen in spite of normal Rock2 expression. In the absence of Rock1, we observe delayed maturation of mast cells, reduced growth in response to cytokine stimulation and impaired migration and adhesion on fibronectin via β1 integrin's and defects in degranulation. In a model of passive cutaneous anaphylaxis, we see reduced responses in mice lacking Rock1 as well as in mice treated with ROCK inhibitor fasudil. These functional changes are associated with reduced activation of AKT and ERK MAPKinase as well as LIMK, as assessed by the phosphorylation of its substrate cofilin. Importantly, deficiency of Limk1, Limk2 or both impaired the growth and maturation of bone marrow derived mast cells. Thus, Rock1/Limk1/2 pathway plays an essential role in regulating diverse functions in mast cells.

While the role of ROCK in non-hematopoietic cells utilizing dominant negative approaches and pan ROCK inhibitors has been well described; recent studies utilizing isoform specific deletion mutants of ROCK have begun to shed some light into how these isoforms contribute to blood cell development and functions. To this end, a role for Rock1 in inflammatory cell migration using *Rock1*−/− mice has been described [28]. An unanticipated role for Rock1 in negatively regulating the recruitment and migration of primary macrophages and neutrophils *in vitro* and *in vivo* in the context of whole organism was also shown [28]. Specifically, enhanced migration of both Rock1 deficient macrophages and neutrophils in response to multiple stimuli was documented. The observed defects were not attributed to changes in the maturation and expression of β1 integrins or to the compensation by Rock2. At the biochemical level, Rock1 was shown to regulate the intracellular levels of phosphatidylinositol (3, 4, 5)-triphosphate/AKT in response to receptor activation by regulating the phosphorylation, degradation, stability and activity of tumor-suppressor protein phosphatase and tensin homolog (PTEN) [28]. Thus, it appears that at least in macrophages, Rock1 plays an important role in regulating the stability and activity of PTEN. Importantly, deregulation of this pathway in Rock1-deficient cells results in enhanced recruitment of macrophages *in vivo*. In the context of a different blood lineage, deficiency of Rock1 in a model of oxidative stress showed rapid and enhanced recovery of red blood cells from hematopoietic stem/progenitor cells in the bone marrow lacking Rock1, in part by regulating the expression/phosphorylation of p53 [29]. These findings are consistent with the fact that loss of p53 in p53-deficient mice also results in enhanced erythroid cell recovery following an oxidative challenge [30]. Taken together, studies described in Rock1-deficient mice, thus far, suggest that Rock1 may play an essential role in negatively regulating the survival of multiple hematopoietic lineages in the context of both inflammatory as well as oxidative stress in myeloid and erythroid cells, respectively, in part by regulating the activation of tumor-suppressor genes such as PTEN and p53. Our current studies in mast cells suggest that the mechanism(s) by which Rock1 impacts macrophage and erythroid cell functions is not the same as how Rock1 regulates mast cell development and functions. Our findings show that Rock1 is a positive regulator of mast cell functions including adhesion, migration and degranulation in part by regulating the activation of LIMK. Thus, Rock1 is likely to regulate cellular functions based on the presence and activation of downstream substrates in specific cell types.

Our biochemical findings suggest that downstream from Rock1, LIMK activation as assessed by phosphorylation of cofilin is significantly altered in mast cells. LIMK is a key regulator of actin cytoskeleton. They phosphorylate and inactivate cofilin. Given that cofilin plays a prominent role in promoting actin depolymerization and that active (unphosphorylated) cofilin induces severing of actin filaments and contributes to cell migration, cell cycle and differentiation, we sought to assess the role of both Limk1 and Limk2 [24]. We show that both Limk1 and Limk2 play an equally important role in regulating the maturation and growth of mast cells. Given our findings in a model of anaphylaxis and recent studies showing an essential role for ROCK in an allergic airway disease model in which mast cells contribute significantly [31, 32], targeting LIMK using small molecule inhibitors may be a prudent therapeutic option.

## MATERIALS AND METHODS

### Cytokines, antibodies and reagents

Recombinant murine interleukin-3 (IL-3) and stem cell factor (SCF) were purchased from Pepro Tech, (Rocky Hill, NJ). Allophycocyanin (APC)-conjugated KIT antibody, Phycoerythrin (PE)-conjugated FcεR1 antibody, PE-Annexin V antibody and 7-Amino actinomycin D (7-AAD) were purchased from BD Biosciences (San Jose, CA). Rabbit anti–Rock1 and anti-Rock2 antibodies were purchased from Santa Cruz Biotechnology (Santacruz, CA). Rabbit anti-MYPT1 antibody was purchased from Upstate (Lake Placid, NY). Rabbit anti-phospho-cofilin, anti-phospho-AKT, anti-phospho-ERK, anti-cofilin, anti-AKT and anti-ERK1/2 antibodies were purchased from Cell signaling Technology (Beverly, MA). Retronectin was purchased from Takara (Madison, WI). Iscove's Modified Dulbecco's Medium (IMDM) was purchased from Invitrogen (Carlsbad, CA). [^3^H] Thymidine was purchased from PerkinElmer (Boston, MA).

### Mice

Mice deficient in Rock1, Limk1, and Limk2 have been previously described [33–35]. All mice were maintained under specific pathogen-free conditions at the Indiana University Laboratory Animal Research Center (Indianapolis, IN). All animal procedures were conducted in accordance with the Guidelines for the Care and Use of Laboratory Animals and were approved by the Institutional Animal Care and Use Committees (IACUCs) at Indiana University School of Medicine.

### Generation of bone marrow derived mast (BMMC) cells

To generate BMMCs, low density bone marrow cells (LDBMCs) from WT and various knockout mice were cultured in the presence of IL-3 (10 ng/mL) for 3–4 weeks. These *in vitro* generated BMMCs were used to perform *in vitro* functional and biochemical experiments described here.

### Mast cell maturation

Maturation of BMMCs from WT and various knockout mice was analyzed by examining the expression of KIT and IgE receptor using flow cytometry as described previously [36]. BMMCs (1 × 10^6^) were blocked with 10% rat serum in phosphate buffered saline (PBS) containing 0.2% bovine serum albumin (BSA) (Sigma, St Louis, MO) for 30 min at 4°C. After blocking, cells were incubated with 1 μg of anti-APC-KIT and anti-PE-FcεR1 antibodies for 30 min at 4°C. Cells were washed to remove unbound antibodies with PBS containing 0.2% BSA and analyzed by flow cytometry (Becton Dickinson, San Jose, CA).

### Proliferation assay

Proliferation was assessed by conducting a thymidine incorporation assay as described previously [37, 38]. Briefly, cells were washed and starved in IMDM containing 0.2% BSA without serum or growth factors for 6 to 8 hours. Cells (5 × 10^4^) were plated in replicates of four in a 96-well plate in 200 μL complete medium either in the absence or presence of the indicated growth factors. The cells were cultured for 48 hours and subsequently pulsed with 1.0 μCi (0.037 MBq) [^3^H] thymidine (PerkinElmer) for 6 hours and harvested using an automated 96-well cell harvester (Brandel). Thymidine incorporation was assessed as counts per minute (CPM).

### Adhesion on fibronectin

*In vitro* integrin-mediated adhesion assay was conducted as previously described [39]. Briefly, flat-bottom 96-well polystyrene plates (BD Biosciences) were coated with 20 μg/mL retronectin in PBS for 1 hour at 37°C. Wells were washed twice with PBS, incubated with 20 mg/mL bovine serum albumin (BSA) for 1 hour at 37°C for blocking nonspecific sites, and again washed twice with PBS. To examine cell adhesion on the coated surface, 1 × 10^5^ cells were added to each well and incubated at 37°C for indicated times. At the end of the incubation, unbound cells were removed by aspiration carefully, and wells were washed twice with cold PBS. Adherent cells were fixed with 3.5% formaldehyde and stained with 0.1% crystal violet. The stain was eluted with 10% acetic acid, and absorbance was determined at 600 nm using a microplate reader (Spectramax 250; Molecular Device, Sunnyvale, CA).

### Integrin-mediated migration assay

*In vitro* haptotactic migration assay was conducted as previously described [39]. Briefly, the transwell filters (8 μM pore filter; Costar, Boston, MA) were coated on the underside with 20 μg/mL retronectin (RN) for 2 hours at 37°C, and rinsed twice with PBS containing 2% BSA. The RN-coated filters were placed in the lower chamber containing 500 μL complete medium with or without SCF (100 ng/mL). BMMCs (2.5 × 10^5^) were re-suspended in 100 μL IMDM and allowed to migrate toward the underside of the top chamber. After 4 hours of incubation at 37°C, non-migrated cells on the upper side of the chamber were removed and the migrated cells attached to the bottom surface of the membrane were stained with 0.1% crystal violet, 0.1 M borate, pH 9.0, and 2% ethanol for 5 minutes at room temperature. The numbers of migrated cells per membrane were counted in 10 random fields with an inverted microscope using X20 objective lens.

### Mast cell degranulation assay

Mast cell degranulation was analyzed as described previously [40]. BMMCs from WT or *Rock1−/−* mice were washed and starved in RPMI containing 0.5% BSA for 6 hours and then resuspended in Tyrode buffer (10 mM HEPES buffer, pH 7.4, 130 mM NaCI, 5 mM KCI, 1.4 mM CaCI_2_, 1 mM MgCI_2_, 5.6 mM glucose, and 0.1% BSA). For measuring degranulation in response to IgE, cells were sensitized with 10 mg/mL anti-dinitrophenyl (anti-DNP) IgE mAb SPE-7 (Sigma-Aldrich) for 1 hour, washed twice with 23°C Tyrode buffer, equilibrated in Tyrode buffer to 37°C for 5 minutes, and then treated for 15 minutes with DNP-human serum albumin (Sigma-Aldrich). The degree of degranulation was determined by measuring the release of β-hexosaminidase.

### IgE-mediated passive cutaneous anaphylaxis (PCA)

Passive cutaneous anaphylaxis mouse model was developed as described previously [27]. WT and *Rock1−/−* mice were sensitized with intradermal injection of 20 ng anti-DNP IgE mAb (Sigma-Aldrich) into the left ears, whereas the right ears received saline as control. After 24 hours, mice were challenged by intravenous injection of 100 μg DNP-BSA in 200 μL of Evan's blue dye (1% wt/vol; Sigma-Aldrich). After 30 minutes, an 8-mm ear punch was collected in 300 μL of formamide and incubated at 80°C for 2 hours in water bath. After incubation, the extract of Evan's blue dye was measured by absorbance at 620 nm in a spectrophotometer. To test whether Rho kinase inhibitor fasudil can be used to treat anaphylaxis, we treated WT mice with Rho kinase inhibitor fasudil 30 minutes before challenging the mice with DNP-BSA.

### Western blotting

Western blotting was performed as described previously [37]. Equal amount of protein extracts were separated on 4–20% SDS-polyacrylamide gels. After electrophoresis, the proteins were transferred onto nitrocellulose membranes and nonspecific binding was blocked with 5% nonfat dry milk in Tris-buffered saline containing 0.1% Tween-20 (TBS-T). Membranes were then probed with various antibodies overnight at 4°C on a rocker. After incubation, membranes were washed with TBS-T and incubated with appropriate horse radish peroxidase (HRP)-conjugated secondary antibodies for 1 hour at room temperature. After washing the membranes with TBS-T, the proteins on the membranes were detected using SuperSignal West Dura Luminol / Enhancer solution (Thermo Fisher Scientific, Rockford, IL) and exposing the membranes to X-ray film.

### Statistics

All graphical data were evaluated by paired Student *t* test, and results were considered significantly different with *P* value < .05. All data are represented as mean + SD or SEM as indicated in Figure legends.
